# PARTICIPATION IN THE COMMUNITY AMONG PEOPLE WITH AND WITHOUT DEMENTIA: DESTINATIONS AND PERCEPTIONS OF CHALLENGES

**DOI:** 10.1093/geroni/igz038.2850

**Published:** 2019-11-08

**Authors:** Habib Chaudhury, Tanveer Mahal, Kishore Seetharaman

**Affiliations:** 1 Simon Fraser University, Vancouver, British Columbia, Canada; 2 University of Sydney, Sydney, New South Wales, Australia

## Abstract

Older adults with dementia face challenges in their outdoor mobility and there are concerns of their not being able to continue going outside for everyday activities and social participation. The focus of this study was to identify patterns of visits to community destinations and activities, and perceptions of risks. Interviews were conducted with 59 adults (aged 54-84) with (n=29) and without (n=30) dementia using the Participation in ACTivities and Places OUTside the Home (ACT-OUT) questionnaire in Vancouver, Canada. Findings indicate that participants with dementia had abandoned visiting a few places over time (e.g., bank, cemetery, buildings of worship), whereas there were no change in participation in taking transit to destinations such as supermarkets, entertainment and cultural places. However, in some cases, companions or partners of persons with dementia indicated that they were prone to getting anxious when left alone in public places and were at high risk of getting lost.

